# Don’t make cache too complex: A simple probability-based cache management scheme for SSDs

**DOI:** 10.1371/journal.pone.0174375

**Published:** 2017-03-30

**Authors:** Seungjae Baek, Sangyeun Cho, Jongmoo Choi

**Affiliations:** 1 KIOST, Ansan, Gyeonggi, South Korea; 2 Samsung Electronics, Hwaseong, Gyeonggi, South Korea; 3 Dankook University, Yongin, Gyeonggi, South Korea; Universita degli Studi di Catania, ITALY

## Abstract

Solid-state drives (SSDs) have recently become a common storage component in computer systems, and they are fueled by continued bit cost reductions achieved with smaller feature sizes and multiple-level cell technologies. However, as the flash memory stores more bits per cell, the performance and reliability of the flash memory degrade substantially. To solve this problem, a fast non-volatile memory (NVM-)based cache has been employed within SSDs to reduce the long latency required to write data. Absorbing small writes in a fast NVM cache can also reduce the number of flash memory erase operations. To maximize the benefits of an NVM cache, it is important to increase the NVM cache utilization. In this paper, we propose and study *ProCache*, a simple NVM cache management scheme, that makes cache-entrance decisions based on random probability testing. Our scheme is motivated by the observation that frequently written hot data will eventually enter the cache with a high probability, and that infrequently accessed cold data will not enter the cache easily. Owing to its simplicity, *ProCache* is easy to implement at a substantially smaller cost than similar previously studied techniques. We evaluate *ProCache* and conclude that it achieves comparable performance compared to a more complex reference counter-based cache-management scheme.

## 1 Introduction

As circuit, manufacturing, and architectural innovations have led to attractive flash memory-based solid-state disks (SSDs) from a performance-cost perspective, SSDs have become a common storage component in recent computer systems. In terms of cost, the main driver has been smaller feature sizes and multiple-level cell technologies such as multi-level cells (MLCs) and triple-level cells (TLCs).

However, as the flash memory stores more bits per cell, the performance and reliability of the flash memory will be substantially lower than when storing fewer bits per cell. To address this problem, a fast non-volatile memory (NVM-)based cache is widely used in modern SSDs.

Any kind of NVM devices can be used as a cache for SSDs. For example, phase-change memory (PCM) or spin transfer torque magnetoresistive RAM (STT-MRAM) can be used as an NVM cache memory. Besides, some portions of a flash memory can be exploited as an NVM cache by applying a special mode that reduces the number of bits stored per cell (e.g., TLC → MLC or MLC → SLC) [1].

An NVM cache can enhance the performance of SSDs by reducing the long latency to write data. Furthermore, absorbing small writes with a fast NVM cache can also reduce the number of flash memory erase operations by ensuring that garbage collection targets behaving more uniformly, thereby prolonging the lifetime of the flash memory.

Regardless of what NVM device is used, developing an efficient cache-management scheme is first essential for maximizing the NVM cache utilization. More frequently written data (i.e., hot data) should be classified and maintained in the cache with minimum space and computational overhead. Increasing the amount of hot data maintained within the cache leads to higher performance and longer lifetime.

In order to maintain a higher hit ratio, and therefore maximize the cache utilization, various mechanisms have been proposed for SSDs [2–9]. However, these schemes require either high computational overhead or significant memory space, both of which significantly affect the cost of the storage system and mitigate the effectiveness of the cache.

This paper investigates a data caching management scheme that exploits the characteristics of a multiple-level cell flash memory. Based on the concept that frequently written hot data will eventually enter the NVM cache with a high probability, and that infrequently used cold data will not enter NVM cache easily without any extra work, we introduce probability based decision making, which is called ProCache. ProCache is fairly simple and can be implemented practically in a real system because it does not require any complex data structures or extensive computations—just throw the dice!

We first show sensitivity analysis studies by performing simulations using the block traces that are collected from real machines. Then, for comparison purpose, we implement an SSD emulator including our proposed scheme along with relevant caching techniques. The results obtained from various perspectives show that the proposed scheme efficiently maintains a high hit ratio, and therefore enhances the overall performance over time and the storage ages. Specifically, ProCache exhibits a performance of up to 16.2%, which is on average a 7.6% better performance, and efficiently decreases the number of write operations by 19.3% and erase operations by 14.0%.

The remainder of this paper is organized as follows. In Section 2, we discuss some related work on this research. In Section 3, we explain the algorithm of the proposed scheme, while in Section 4, we discuss the trace-driven analysis results. Then, in Section 5, we describe the experimental setup and performance comparison results using an SSD emulator. Finally, we conclude this paper in Section 6.

## 2 Background

In their early work of 1997, Chiang et al. proposed a flash memory server (FMS) in order to reduce the number of erase operations in flash memory [2]. While FMSs exhibit good hot and cold data classification performance, they require a large amount of memory space because they must retain the last access time information of all LBAs. Unlike FMS, our probability-based scheme does not require recording for each set of LBA information for hot/cold data classification.

In a later work, Chiang et al. propose a new data reorganization method called dynamic data clustering (DAC) [3]. DAC dynamically clusters data not only during segment cleaning, but also during data updates. Our approach is fundamentally different from DAC, and does not require any substantial data management operations.

Chang et al. proposed a two-level LRU list scheme that employs a hot LBA list and a candidate list [4]. This two-level LRU scheme consumes less memory than FMS; however, it incurs other problems. The performance of hot data identification is totally dependent on the sizes of the lists. In other words, a small hot list can save memory space, but its prediction accuracy degrades because hot data may be demoted to the candidate list or may even be evicted from the candidate list. Moreover, this scheme incurs relatively high computing overheads to maintain LRU information. Our scheme does not maintain any LRU information or separate lists to classify LBAs.

Chang considers the size of write requests in his hot/cold prediction scheme [5]. We find that our approach and this size-based prediction approach are orthogonal. The write request size may provide us with useful hints. For example, depending on the size, we may apply different probability values when determining whether to insert the data into the cache.

There are schemes that track hot/cold data using compact tables. Hsieh et al. adopt *K* independent hash functions to hash a given LBA into multiple entries of an *M*-entry hash table to track the write count of the LBA [6]. Whenever a write is issued to the FTL, the corresponding LBA is hashed simultaneously by the hash functions. Each corresponding table entry (counter) to which the hash values point is incremented by one to reflect the fact that the LBA is written. In order to track the temporal access pattern changes, the counters are decayed (by dividing each entry by two) periodically. This approach can be fairly accurate, but is too expensive for application in a practical system with a large capacity.

In another study, Park and Du adopt a set of *V* independent Bloom filters (BFs) and *K* independent hash functions [7]. The weaknesses of the multiple hash function approach are: (1) the memory requirements of tables increase as the capacity of the storage device increases, and (2) computing *K* hash values and manipulating multiple tables may incur additional computational overhead.

Jung et al. propose a new hot/cold identification scheme that uses process identification (PID) as a hot/cold indicator [8]. The weakness (or strength) of this scheme is that it utilizes the information in the OS, which is generally not available inside a storage device. Our scheme does not require the OS level information in the core algorithm.

Finally, Kim et al. argue that the hot data (e.g., file system metadata) may be effectively compressed, while the cold data (e.g., multimedia data) may not be because they are already encoded [9]. In itself, the merit of this scheme is limited owing to the required computation overhead (unless compression is always performed for the purpose of saving storage capacity). It is also unclear whether the proposed method is more accurate than other previous ideas. In addition, our scheme uses the frequency of data accesses, which we believe is more general and accurate.

## 3 Probability-based cache management

**Fig 1:**
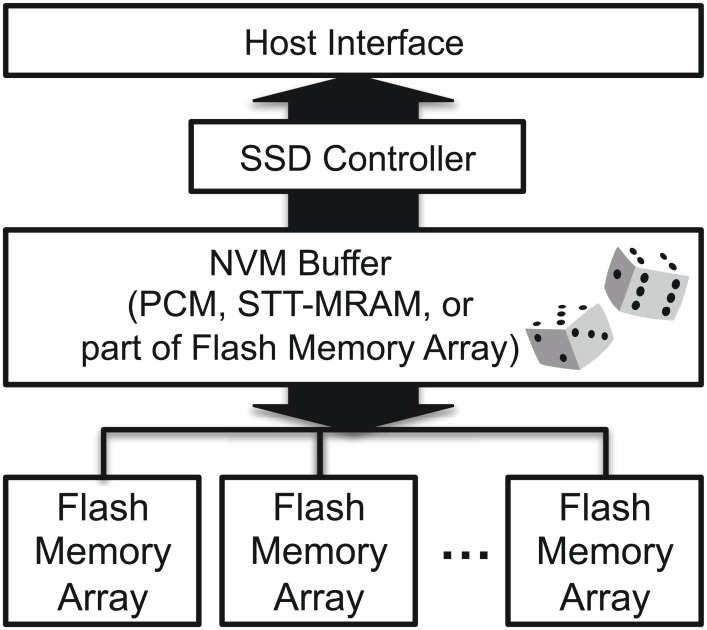
Storage system internal key components.

Fig 1 depicts the key components of the typical flash memory-based storage system, a host interface, an SSD controller, an NVM cache, and a NAND flash memory array. To handle read and write requests that are issued from an operating system via a host interface, an SSD controller reads or writes data according to the request type.

We propose a probability-based cache-management algorithm, which we call ProCache. ProCache is built on the simple probabilistic principle that repeated trials with a small probability of success will eventually result in success.

At the heart of our algorithm is the probability-based data classifier. In its baseline form, the input to the classifier includes *p*(0 < *p* ≤ 1), which is the probability of declaring that the current input is hot, and *c*, which is the cut-off size that determines whether a write is “too” large. One may expand the notion of *p* to a vector, p* = (*p*_0_, *p*_1_, *p*_2_,…, *p*_*N*−1_). In this case, given the request size of *C*, $p_{\left\lceil {\text{log}}_{2}c\right\rceil }$ is selected for the test. In this way, one may eliminate the second parameter *c*, or, depending on the choice of *N*, 2^*N*−1^ may become the new *c*.

The goal of designing a ProCache is to prove that the proposed probability-based NVM cache management concept can be implemented practically in a real system. Upon receiving a new write request, ProCache works like the Algorithm 1. As shown in the Algorithm 1, the proposed ProCache is fairly simple and easy to implement.

**Algorithm 1** ProCache algorithm

**if** the requested block is already in the cache **then**

 It is hot data. Update corresponding NVM cache data.

**else**

 **if** the request size ≥ *c* **then**

  It is cold data. Send it to the NAND array and invalidate any data in the

  NVM cache that overlaps with the request.

 **else**

  Generate a random number *r*, 0 ≤ *r* ≤ 1.

  **if** *r* < *p* **then**

   The write request passed the probability test and is considered hot.

   Insert it to the cache and invalidate any data in the cache that

   overlaps with the new data.

  **else**

   The data is cold, and send it to the NAND array and invalidate any

   data in the NVM cache that overlaps with the request.

  **end if**

 **end if**

**end if**

On the other hand, its effectiveness is expected to depend largely on the choice of *p* and *c*. Our preliminary study using a trace-driven simulation methodology shows that the proper choice of *p* and *c* is important for the above algorithm to perform well. First, we examine the algorithm theoretically before analyzing the initial experimental results.

The average number of trials needed to make an LBA “hot” is, given *p*, follows the *geometric distribution*:

$$\begin{array}{c} p+2\cdot \left(1-p\right)\cdot p+3\cdot {\left(1-p\right)}^{2}\cdot p+\ldots \\ =\sum_{i=1}^{\infty }i\cdot {\left(1-p\right)}^{i-1}\cdot p \end{array}$$ (1)

The above series boils down to 1/*p*. That is, if *p* = 0.1, the expected number of trials is 10 before an LBA is considered hot. If *p* = 1, the first trial will declare the LBA to be hot. In turn, in order to verity that hot data will usually have repeated access count of *R* on average, we can set *p* to be simply 1/*R*. Likewise, we can compute the probability that a “cold” LBA, which has *K*-th accesses, is not identified as “hot.” This probability is equivalent to one less the probability of this address being declared hot within *K* trials. That is,

$$\begin{array}{c} 1-(p+\left(1-p\right)\cdot p+{\left(1-p\right)}^{2}\cdot p+\ldots \\ +{\left(1-p\right)}^{K-1}{\cdot p)=\left(1-p\right)}^{K} \end{array}$$ (2)

When *p* = 0.1 and *K* = 3, the above probability is 0.729, adn when *p* = 0.05 and *K* = 3, the above probability is 0.857. The above result implies that with a sufficiently small *p*, the probability of having cold data enter, and thus pollute, the cache is not high. In other words, cold data are kept off the cache with a high probability of over 72% or 85% when *p* = 0.1 and *p* = 0.05, respectively (when *K* = 3).

In summary, the proposed probability-based hot data classification algorithm is simple and easy to implement. As long as there is a wide gap in the access frequencies between hot data and cold data, the probability-based classifier with a small *p* will effectively distinguish them. If it is important to quickly classify hot data, we need to have a reasonably large *p*. If preventing cache pollution is important, we need to keep *p* small.

For comparison purposes, we introduced an off-line optimal cache-management algorithm. Basically, replacement policies identify the block that will be used in the furthest future for a better cache memory utilization. Belady’s off-line algorithm discards the information that will not be needed for the longest time [10]. Even though off-line algorithms are the most efficient cache-replacement algorithms, it is not able to adapt them in real systems because it is impossible to predict the future. We note that off-line algorithms are not for real implementation, and are developed to expose insights about what the on-line ProCache algorithm can or cannot achieve.

Our off-line algorithm is derived from the well-known Belady’s algorithm. We add the notion of “bypass” to Belady’s algorithm: If the newly requested data will not be needed again for a time longer than all the information already in the cache, we bypass the caching of this data; otherwise, we follow Belady’s algorithm.

In the example shown in Table 1, we consider four successive requests for a given current cache status. Each pair of numbers (such as (1,4)) represents (LBA,next), where LBA is the logical block address of a request, and next is the logical time when the same address is next requested in the future. In the first case, read (1,5). The request hits in the cache (LBA 1 is in the cache) and the next access time of the cache entry is updated (from 4 to 5) based on the next access time of the new request. In the second case, read (5,8). Then, the request misses in the cache. Moreover, because this request is a read, we do not cache the logical block in the cache. Next, for the new request, write (1,6). There is a hit in the cache and the corresponding cache entry is updated with the next new access time. Finally, the request write (5,8) misses in the cache. Because this request is a write, we need to determine whether it should be inserted into the cache. Using the original Belady’s algorithm, this request is inserted and the logical address 4 is evicted because its next access time (7) is the largest of all that are in the cache. On the other hand, if cache bypassing is enabled, the logical address 5 is not inserted into the cache because its next access time (8) is larger than that of any address in the cache.

**Table 1:** An example showing the initial cache content and how a new request changes the cache content.

Cache content	{(1,4), (2,5), (3,1), (4,7) }
New request	corresponding action
Read (1,5)	read hit; update (1,4) to (1,5)
Read (5,8)	read miss; cache is NOT updated
Write (1,6)	write hit; update (1,5) to (1,6)
Write (5,8)	write miss; if no-bypassing,
	evict (4,7) and insert (5,8);
	if bypassing, bypass (5,8)

Note that in our off-line algorithm, we label each access in the trace with the next access time information before we process the requests in sequence from the beginning according to the method described above. As such, the off-line algorithm utilizes the knowledge about future references to a given address.

## 4 Trace-driven analysis results

**Fig 2:**
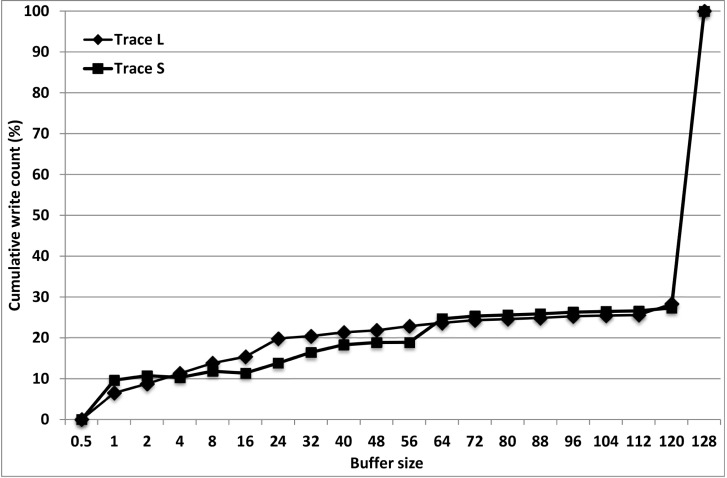
Cumulative write count.

We used two traces for experiments, as summarized in Table 2. Traces were collected from a working desktop PC using the Windows PerfMon facility. As shown in the table, traceS is relatively short (a few days’ amount) and traceL is relatively long (at least a week’s amount). The cumulative write count of traceL is shown in Fig 2. Please note that the cumulative write count of traceS is very similar to that of traceL. Note that traceS is from a typical office computer and traceL is from one that is for development. The main metric that we used in this section is the write hit ratio, which is the ratio of the number of write transactions that hit in the cache to the total number of write transactions.

**Table 2:** Basic characteristics of the two traces used in experiments.

Name	traceS	traceL
# transactions	334,135	6,803,769
Reads vs. Writes	64:36	47:53
Total write volume	10.5 GB	49.8 GB

### 4.1 Comparison of off-line and ProCache algorithms

**Fig 3:**
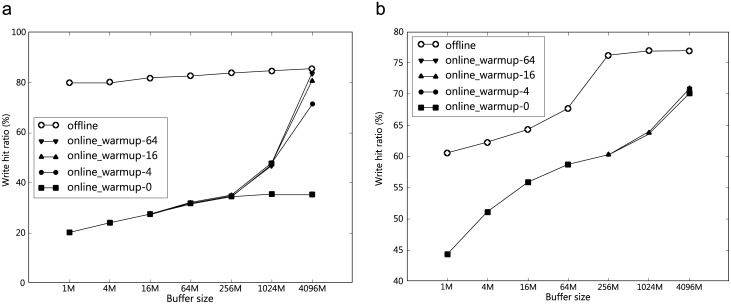
Off-line vs. ProCache algorithms. *p* = 0.5, *c* = 64 KB, (a) traceS, (b) traceL.

Fig 3 shows the performance of the off-line algorithm (“off-line”) and the performance of the ProCache algorithm (“on-line”) as we vary the number of warm-up iterations. Warm-up refers to running the given trace through the algorithm to “warm up” the write cache. We consider warm-up because the traces that we study correspond to only several days of storage traffic. Then, we repeated warm-ups to artificially amplify the traffic. We did not measure the performance of the ProCache algorithm during the warm-up iterations. We studied the effect of warm-up separately in Section 4.4, and we make several observations. ProCache is a write cache as described in Section 3. To quantitatively evaluate the performance of the scheme, we measure the cache hit ratio. The hit ratio is calculated by dividing the write hit count by the number of requests, and is between 0∼100%.

- The hit ratio of the *off-line* algorithm is high, from the smallest to the largest cache size that we examined, and the difference between the smallest and the largest cache sizes was small. This implies that the working set size is fairly small. The write hit ratio was as high as 80% or higher.
- For all cache sizes, *off-line* performs better than the on-line algorithm. *On-line* approaches *off-line* only when the cache is very large (4 GB), and we performed many (16 or more) warm-up iterations. The result suggests that *off-line* places a fairly tight upper bound on the *on-line* performance. Furthermore, if we observe the *on-line* performance for a long period of time, the *on-line* performance (with a high *p*) will converge to that of the *off-line* if we have a large cache. On the other hand, with a relatively small cache, the *on-line* algorithm does not perform as well as *off-line*. It should be noted that the *on-line* algorithm makes a decision regarding caching at the granularity of a request (constrained by *c*), whereas the *off-line* algorithm keeps track of the access information with a 4-KB block granularity.

### 4.2 Effect of *p*

**Fig 4:**
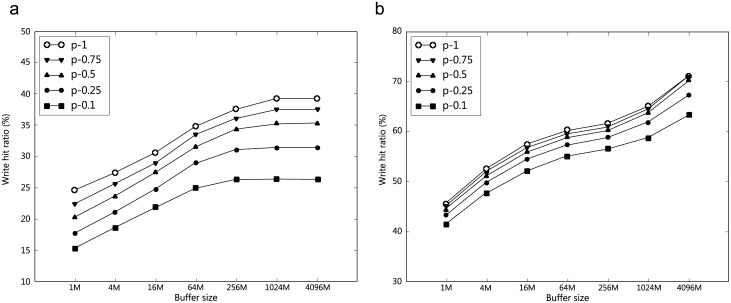
Effect of *p* on write hit ratio: *c* = 64 KB. (a) traceS, (b) traceL.

As was previously discussed, *p* is the main parameter employed in the probability-based hot data classification algorithm. Fig 4 depicts the effect of *p* on the write hit ratio for both traces.

- In general, with respect to the examined metric (write hit ratio), a large *p* performed better than a small *p*.
- The highest write hit ratio that was observed was about 38% (traceS) and nearly 70% (traceL).
- The difference between the smallest and the largest *p* examined (0.1 and 1) increases with the cache size for both traces. Interestingly, the gap is narrower with traceL than traceS. We find that if we study for a much longer time span, a small *p* will perform fairly close to a large *p*, especially when the cache size is small.
- Based on the result, if the available cache size is large, an interesting approach may be to use a large *p* to capture the majority of the initial write data. Then, we adjust *p* as the cache occupancy approaches 100%.

### 4.3 Effect of *c*

**Fig 5:**
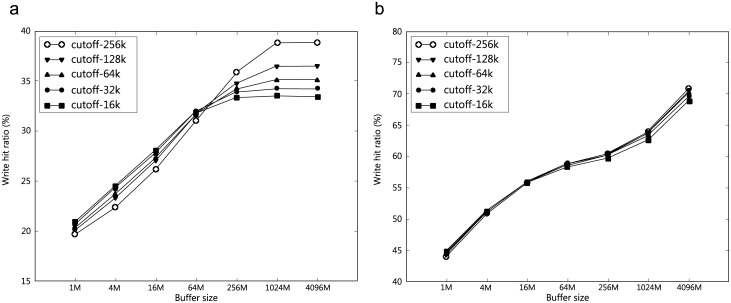
**Effect of *c* on write hit ratio:** *p* = 0.5, (a) traceS, (b) traceL.

Fig 5 shows how the write hit ratio varies with the cache size and *c*.

- A large *c* does not perform as well as a small *c* if the cache is small. Conversely, a large *c* performs better than a small *c* when the cache is large.
- With a large *c*, there appears to be more cache pollution (using large data). It has been reported that large data are reused less than small data.
- The result reveals that *c* is an important parameter for traceS. However, the impact of *c* becomes less significant with traceL, which is much longer than traceS. For traceL, *p* appears to be more important than *c* (Figs 4 vs. 5).
- The result also suggests that the write hit ratio is determined primarily by small writes (32 KB or smaller) if one observes write traffic for a sufficiently long time (traceL).

### 4.4 Effect of warm-up

**Fig 6:**
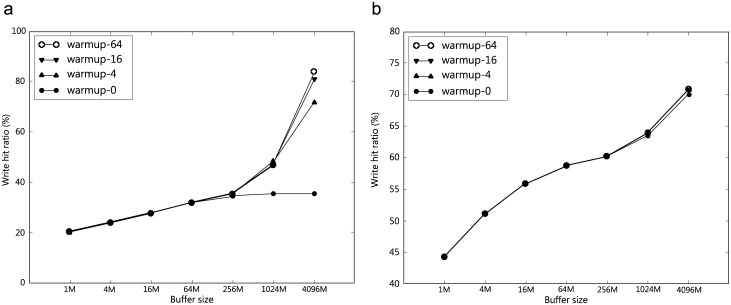
**Effect of warm-up on write hit ratio:** *p* = 0.5 *c* = 64 KB, (a) traceS, (b) traceL.

While our traces are non-trivial, they only correspond to disk usage for a few days. As such, our trace-driven simulation method may not fully expose the characteristics of a cache-management scheme (as indirectly implied by the results in previous sections). In order to quantify the potential limitation of the trace-driven simulation methodology, we studied the effect of “warm-up” of the write cache by running the same trace multiple times before measuring the result. The proposed experiments will artificially increase the number of writes. Fig 6 reports four results: warmup-N has N warm-up runs before we perform the final measurement run. We observe that:

- With warm-up, the write hit ratio increases and the warm-up effect is more visible with a large cache and when the trace is small. This result follows from the fact that repeated warm-ups increase the number of writes seen by the cache, implying that the probability that write data will be kept in the cache is higher.
- With many warm-ups (16 or 64), the write hit ratio becomes fairly high for traceS, and this is partly because the write requests that occur for the first time in the trace may hit in the cache (because the cache has been warmed up artificially).
- Warm-up has little effect for traceL because this long trace has many requests. Moreover, with the high probability considered in the experiment (*p* = 0.5), there is a high feasibility that most repeated writes are all cached.
- Our result suggests that we should be extremely careful when using a relatively short trace to study the behavior of a storage device. Still, the benefit of using traces is that we can perform deterministic experiments.

## 5 Experimental results using real workload

### 5.1 Setup and workload

The above-mentioned results motivate us to develop a new on-line emulation infrastructure. This infrastructure is expected to help us overcome a few shortcomings of the existing trace-driven simulation approach. With trace-driven simulation, it is hard to predict the actual execution time because the interaction of the SSD performance and the system behavior is not captured (i.e., the system condition is different between the time when the trace is collected and when the trace drives the simulation). In addition, trace lengths are typically small in order to conserve the storage space to keep the traces. Lastly, the storage content (e.g., the amount of data that we have at the time of simulation, and the location of the file system metadata) is typically not modeled. The proposed infrastructure is different from the trace-driven simulation method, and is potentially more powerful because:

- It emulates the internal components of the target SSD device while the system runs real workloads;
- Using powerful processors and high-bandwidth main memory in modern commodity PCs, we can model not only the FTL activities but also incorporate realistic timings of key events (e.g., erase operation and channel contention); and
- It becomes possible to create a snapshot of the storage content so that we can repeat emulations predictably under real workloads.
- For a fair and fast comparison, we may emulate many different SSD configurations (e.g.,different cache sizes) simultaneously without having to consider the repeatability of the study.

The new on-line emulation infrastructure allows us to run significantly longer and larger workloads (compared to the trace-driven simulator we developed), and it increases the productivity and reliability of the experiments. Furthermore, it will help us determine the effect of the run-time performance of the on-line algorithm. The trace-driven simulation methodology is limited, and cannot accurately predict the performance impact of the internal working of the SSD on the overall system performance.

We have built our emulation infrastructure on a real hardware system with a dual-socket server having two quad-core Xeon (E5620) and 192 GB of DDR3 DRAM capacity. This large memory capacity allows us to model a realistic SSD device larger than 160 GB, and to employ large realistic workloads that combine the OS and multiple co-scheduled applications.

**Fig 7:**
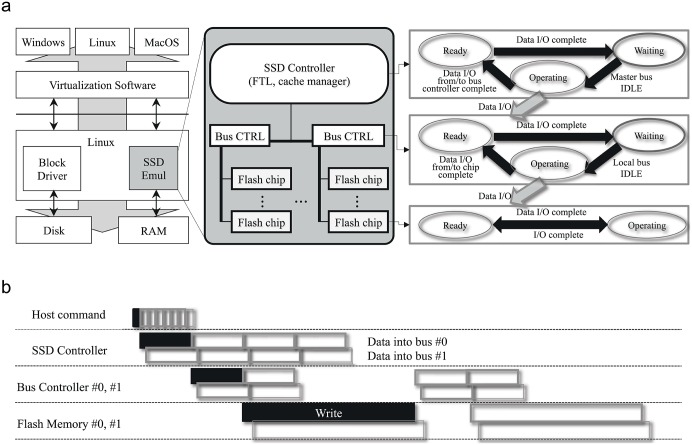
SSD emulator. (a) SSD emulator internal architecture, (b) Example of operation timing diagram for write operation.

To the system, our proposed emulation environment is a completely functional SSD storage device, as shown in Fig 7a. It is a driver running in the kernel space that provides a pseudo block device to the host. The environment is a pure software driver, therefore no other hardware is required. Accordingly, the system cannot distinguish our emulated device from a physical device. This implies that with proper virtualization software, we can run workloads on a Linux or Windows (or any other OS supported by the virtualization technology of choice) guest with no modification to the OS itself.

It internally emulates the SSD controller, bus, bus controller, and flash chips, and allows the user to configure the parameters of each component. Specifically, it enables the user to configure the size of the command queue, type of FTL (page, block, and hybrid), number of buses, bus speed, bus controller delay, and various parameters for each flash memory chip including the *page* size, *spare* area size, number of *page*s per *block*, number of *block*s per chip, number of chips, and read/write/erase operation overhead [11–13].

At the same time, the timing behavior of the target SSD device is controlled by user-defined parameters, thus giving us emulation capabilities. The emulated SSD controller, bus controller, and each flash memory chip are emulated separately by using the concept of the state machine.

Fig 7b shows an example of the operation timing diagram for the write operation. In this example, assume that the emulator has eight command queue buffers, two local buses, and two chips per bus.

When the emulator receives a write command from a host, the request is queued in the command buffer, and is serviced in a first-in first-out (FIFO) manner when the status of the SSD controller module becomes *Ready*. Then, it changes the controller’s status to *Waiting*, and copies actual data to the SSD controller module, where the controller executes the FTL function during the *Waiting* state. After that, the controller’s status becomes *Operating*, and it copies the data to the appropriate local bus when the local bus’s status is *Ready*. Before changing each component’s status, we artificially emulate the timing overhead as designated by the user.

For the experiments, we set the SSD emulator geometry related parameters, as shown in Table 3, and we used flash memory timing parameters based on a typical TLC flash memory chip specification. Then, we implemented our ProCache into an SSD emulator. The current implementation of ProCache uses a simple LRU-replacement policy. It means that ProCache uses the probability based cache-entrance policy and LRU based cache replacement policy. The LRU policy and its variants base their replacement decision on the age of the references, while the LFU policy and its variants base their decision on the frequency of occurrence of the references [14]. However, note that the use of different replacement policies does not significantly affect the result because of the characteristics of ProCache. We used the Ext4 file system with a 1-KB block size, which is the default block size.

**Table 3:** SSD emulator parameters.

Parameter	value
Page size	2KB
Pages per block	128
Blocks per chip	4096
Number of chips	32
Number of buses	4
Number of channels	4
R/W/E latency	20us/200us/1.5ms

To compare the performance using a realistic workload, we defined a set of common works, as shown in Algorithm 2, which reflects (a little bit heavy) daily common computer usage pattern. The workload that we used for the experiment consists of four steps. First, we copied the Linux kernel sources from the original directory to a new directory, and we compiled those kernel sources using multiple threads; specifically, we ran 16 threads simultaneously. The storage occupancy of this work is around 6.3 GB. Then, we cleaned the source tree. Secondly, we copied two DVDs and one CD file to a new directory four times. To do this task, we required about 25.2 GB of storage space. Third, we downloaded 2,000 web pages to a local directory. This step required a storage space of about 130 MB. Finally, we removed the newly created DVDs, CDs, and web pages. This workload shows various potential write patterns, that is, a mixed-size random pattern for the first step, a long sequential pattern for the second step, and a short sequential pattern for the third step.

**Algorithm 2** Test workload.

**for** i = 0; i < N; i++ **do**

 Kernel source copy and kernel compile (∼ 6.3GB) and clean

 4 x 2 DVDs and 4 x 1 CD copy (∼ 25.2GB)

 2,000 web pages download (∼ 130MB)

 rm -rf DVD/ CD/ webpages

**end for**

### 5.2 Deciding *c*, *p* and cache size

**Fig 8:**
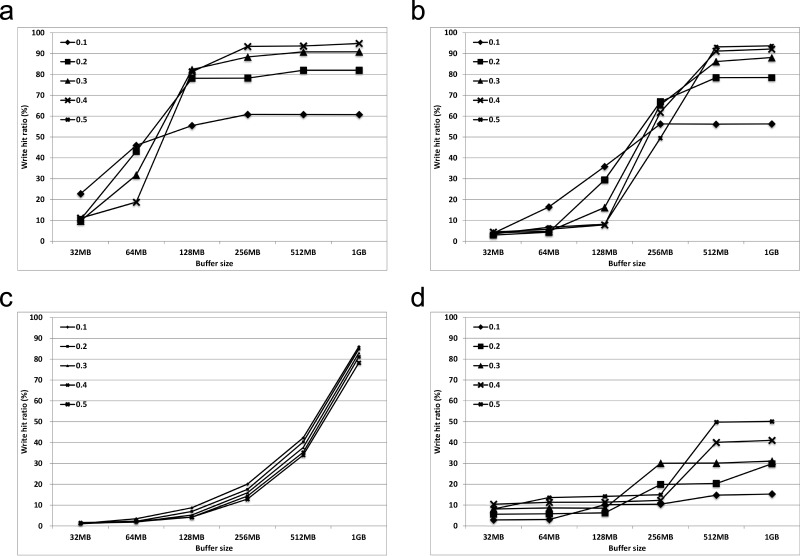
Measured hit ratio when considering only requests that are the same as or less than the cut-off size (warmup-6). (a) *c* = 1*KB*, (b) *c* = 4*KB*, (c) *c* = 8*KB*, (d) *c* = 16*KB*.

**Fig 9:**
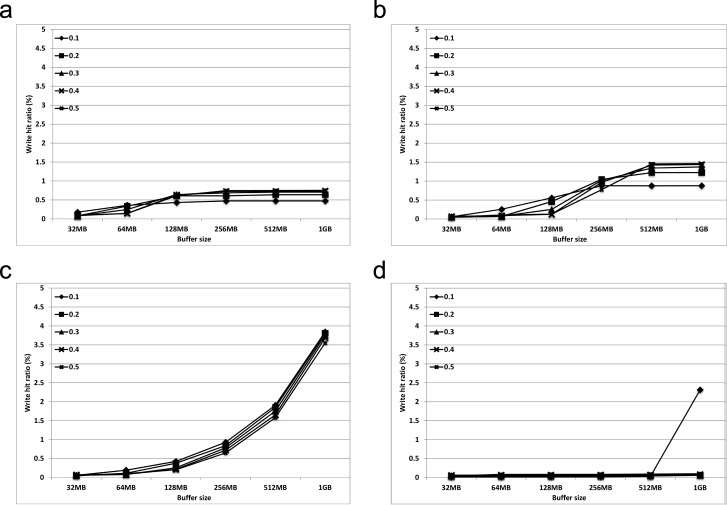
Measured hit ratio when considering all requests (*k* = 6). (a) *c* = 1*KB*, (b) *c* = 4*KB*, (c) *c* = 8*KB*, (d) *c* = 16*KB*.

We first have to determine three parameters, namely *c*, *p*, and the cache size. To determine the appropriate values, we measured the hit ratio of each configuration when the workload shown in Algorithm 2 is executed. Both Figs 8 and 9 show the variation in the hit ratios as we varied *c*, *p*, and the cache size. In Fig 8, we considered only the requests that were the same or less than the cut-off size for calculating the hit rate, while we considered all requests for Fig 9.

The results obtained from a real workload on a real machine confirmed our findings of trace-driven studies.

- For the small *c* (1 KB and 4 KB), a small *p* exhibits a better performance for a small cache size, but a large *p* is appropriate for the larger cache size.
- For the large *c* (8 KB and 16 KB), a large *p* is suitable.
- In the case of a ‘hit’, the data from the cache are used for requests whose size is larger than *c*. Therefore, the hit ratio values of Fig 8 are very high.
- The use of 16 KB for *c* is somewhat large for a real workload.
- The highest hit rate value was obtained when *c* = 8 KB, *p* = 0.1, and the cache size is 1 GB.

Although the hit rate is less than 4% when we consider all of the requests, our cut-off idea significantly affects the performance of the storage system because

- The storage system performance is significantly affected by small random writes [15, 16].
- The *metadata* update of file systems incurs frequent synchronous small write operations, which exhibits extremely hot behavior. For our workload, 24.6% of the total requests were issued by only 1.5% of the small write volume, which was also observed in [17, 18].
- It is extremely important to reduce the number of small write operations, especially in flash memory-based storage systems because of their merge operation overhead [19].

As expected, if there is more cache memory, there may be a greater performance gain. Furthermore, there is a direct correlation between the effectiveness of the proposed idea and the parameters, and the performance of the idea is highly dependent on workloads. However, we need to choose a reasonable cache size. As a result, for the performance comparison experiments, we set *c* as 8 KB, *p* as 0.1, and the cache memory size as 1 GB, which will be described in the next section.

### 5.3 Performance comparisons

In this subsection, we compare our proposed ProCache with the reference counting scheme proposed by Hsieh et al. [6]. The reference counting scheme maintains a saturating counter for each logical block number to be tracked. For comparison purposes, we implemented the pure LRU scheme (denoted as BASE), and the reference counting scheme (denoted as REFCNT) The detailed algorithm used to implement the reference counting scheme is shown in Algorithm 3.

**Algorithm 3** Reference counting scheme.

Initially all *C*_*n*_ have a value 0

Every once in a while (after physical or logical time *t*), decay all *C*_*n*_ (e.g., shift right)

For each write

**if** *C*_*i*_ < *threshold* **then**

 Increment *C*_*i*_

 Write into flash memory

**else**

 Write into an NVM cache

**end if**

For the comparison, we set the SSD emulator parameters for cache memory to be the same as that of the SLC flash memory, while we used the TLC flash memory parameters for the remainder of the main storage area. For both the ProCache and reference counting scheme, we set the cache size as 1 GB and the main flash memory storage size as 32 GB, and for the baseline, we used 33 GB of flash memory.

**Fig 10:**
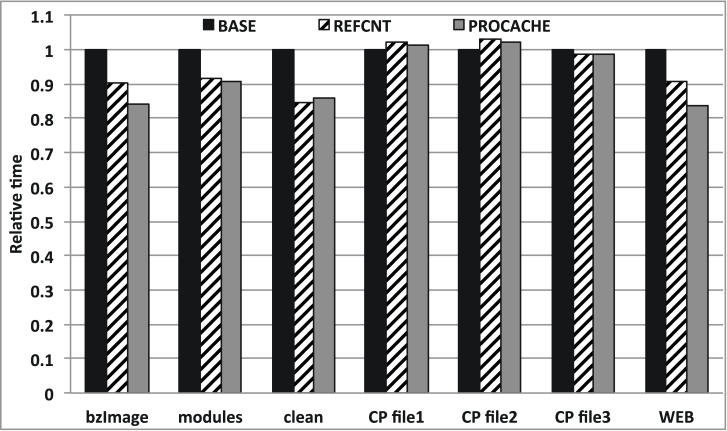
Performance comparison.

We first measured the elapsed time for executing the workload described in Algorithm 2. Fig 10 shows the performance results of the baseline, reference counting scheme, and our proposed ProCache.

ProCache provides up to 16.15% (7.57% on average), better performance than the baseline owing to its efficient cache-management mechanism. In particular, it provides notable performance for workloads that have small random write behaviors, including kernel source compile, clean, and web page downloads. As expected, it is clear that there is no or worse performance gain for the file copy workload because it has a very long sequential cold write pattern. This means that we can further enhance the performance if we can carefully bypass such a long sequential write pattern.

ProCache exhibits a slightly better performance than the reference counting scheme (1.97% on average). Note that the reference counting scheme requires significant RAM space, while ProCache does not. Specifically, the naive reference counting scheme requires 1B for each 512-B block. Therefore, a 512-GB drive requires a RAM overhead of ∼1-GB. The bloom filter can reduce this overhead at the cost of an increased number of memory accesses and false positives. It is very important to reduce the RAM space requirements when applying the proposed techniques to real-world consumer electronics.

**Fig 11:**
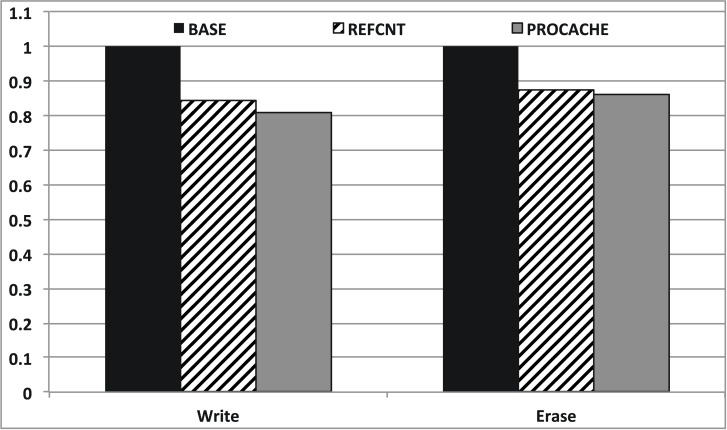
Write and erase count.

Fig 11 shows the results of write and erase count comparison between ProCache and the reference count scheme. Both schemes efficiently decrease the number of write and erase operations, which directly affects the lifetime and performance of the flash memory-based storage system. Specifically, ProCache decreases the number of write and erase operations by 19.3% and 14.0%, respectively. These results explain the reason for which ProCache shows a slightly better performance than reference counting.

## 6 Conclusions

We proposed a simple probability-based hot write data caching scheme. It is simple to realize the proposed scheme owing to its low overhead in terms of RAM usage and computation. The proposed scheme was shown to achieve fairly robust effectiveness at a fraction of the implementation cost needed for other techniques. We first evaluated the scheme using trace-driven simulations. Then, we quantitatively characterized the effect of *c*, *p*, and the cache size. Based on the realistic SSD emulation environment on a live system, we showed the results of the performance comparison. ProCache exhibits a performance that is up to 16.15%, which is on average 7.57% better performance, and it efficiently decreases the number of write and erase operations by 19.31% and 13.99%, respectively.

Based on actual experiments, this paper makes the following contributions:

- Determination of *c*: The main factor is the cache size; if the cache is small, keep *c* small; otherwise, a reasonably large *c* will suffice.
- Determination of *p*: An important factor is the total writable volume to the cache (*c* also affects this). A small *p* of 0.1 appears to be suitable when *c* is 8 KB; a larger *p* of 0.2∼0.4 appears to be suitable when *c* is smaller.
- We found that an SSD emulator with virtualization software support is very useful for the analysis of the internal and external structure of future SSDs. It is very easy to emulate multiple platforms with many configurations simultaneously, and this enables us to closely examine the traffic of systems in a simple manner.

We envision two potential directions for future research. First, we will explore the combination of *p* and *c*. For example, *p* = *P*[*logs*], where P has a decreasing density. Therefore, we can use a different *p* for a different write size (*s*). Second, we will consider dynamically adjusting *p* and *c*. When the replacement rate is high, it may be that *p* is too high and *c* should be decreased.
